# Are They Still Friends? Friendship Stability of Adolescents With Chronic Pain: 1-Year Follow-Up

**DOI:** 10.3389/fpain.2021.767236

**Published:** 2022-02-01

**Authors:** Paula A. Forgeron, Bruce D. Dick, Christine Chambers, Janice Cohen, Christine Lamontagne, Gordon Allen Finley

**Affiliations:** ^1^Faculty of Health Sciences, School of Nursing, University of Ottawa, Ottawa, ON, Canada; ^2^Department of Anesthesia, Dalhousie University, Halifax, NS, Canada; ^3^Children's Hospital of Eastern Ontario's Research Institute, Ottawa, ON, Canada; ^4^Department of Anesthesiology and Pain Medicine, Faculty of Medicine and Dentistry, University of Alberta, Edmonton, AB, Canada; ^5^Pediatrics and Neuroscience, Dalhousie University, Halifax, NS, Canada; ^6^Psychology, Dalhousie University, Halifax, NS, Canada; ^7^Behavioural Neurosciences and Consultation Liaison Team, Mental Health, Children's Hospital of Eastern Ontario, Ottawa, ON, Canada; ^8^School of Psychology, Faculty of Social Sciences, University of Ottawa, Ottawa, ON, Canada; ^9^Department of Anesthesiology and Pain Medicine, University of Ottawa, Ottawa, ON, Canada; ^10^Department of Medicine, University of Ottawa, Ottawa, ON, Canada

**Keywords:** adolescent chronic pain, social functioning and adjustment, friendships, longitudinal, pain-related social outcomes

## Abstract

Most adolescents identify their best friend as their main source of social support. Adolescents with chronic pain (ACP) report the loss of friendships due to pain. Friendships protect against loneliness and depression, yet adolescents with pain experience increased levels of loneliness and depression compared to peers. This longitudinal study examines the friendship stability of dyads that included an adolescent with chronic pain compared to non-pain friendship dyads as well as the factors contributing to a friendship breakup. Eighty-three participants from 61 same-sex friendship dyads across 3 sites participated in a 1-year follow-up survey designed to capture friendship features, indices of social-emotional well-being, pain characteristics, and friendship stability. Chi-square, repeated measures ANOVA, and logistic regression were used to analyze the data. Dyads that included an ACP experienced higher rates of friendship breakup. The shorter length of friendship and having chronic pain predicted a friendship breakup at time 2. ACP continues to experience worse scores on indices of social-emotional well-being that are not predicted with a friendship breakup. Understanding what contributes to positive long-term friendships for those with pain may inform strategies to maintain and improve friendships for those with pain and who experience social challenges.

## Introduction

Chronic pain negatively impacts all aspects of an adolescent's health, including their social health. The WHO describes a child's social health as inclusive of a range of social roles and functions with peers, families, and others (1). Friendships are a vital component of social health, and, by mid-adolescence, most adolescents identify their best friend as their main source of social support (2, 3). Psychosocial factors have been implicated in the prediction of an adolescent with chronic pain over time (4), and adolescents facing peer relationship problems have doubled the risk of developing persistent low back pain (5), indicating that social functioning in adolescents with chronic pain (ACP) has long-term effects.

Limited research on the social functioning of ACP indicates that disruptions to their friendships are common (6). Friendship interactions are reported as negative at times due to pain and they acknowledge friendship loss with pain onset (7). ACP has been found to be selected less often as the best friend and have fewer reciprocal friendships, and they were rated as less likable compared to peers in the same classroom (8). The reasons for friendship disruptions and the losses experienced by ACP include a perceived lack of empathy and disbelief from others (7, 9, 10). However, not all ACPs report difficulty in *making* close friends and self-rate their ability equal to normative data (11). However, it is not known if they are able to maintain these friendships similar to non-pain peers. Of great importance, friendships can be protective for ACP. Positive peer relationships have been linked to more favorable social comparisons for ACP (12), and peer relationship quality moderates the relationship between pain severity and pain-related disability (13). Additionally, some ACPs acknowledge that talking about their pain with friends was easier than talking to their parents (14), illustrating that stable friendships can provide pain-related social support. Moreover, increasing one's peer network and developing more intimate friendships are a hallmark of adolescence. Adolescent friendships provide one with reliable alliance and companionship, and they can buffer negative effects of peer victimization (15, 16). They also protect against loneliness and depression (17), indicating that strong friendships are necessary for healthy adolescent development. Since ACP score being higher on loneliness, depression, and anxiety measures compared to controls and being lower on self-esteemed measures in cross-sectional studies (18, 19), it is critical to understand if and how friendship stability impacts these factors over time.

Conflicting findings are reported in the wider adolescent literature on the importance of same-sex positive vs. negative friendship features as the predictors of friendship stability over time (20–22). Some studies also suggest that a non-clinical degree of internalizing behaviors strengthened friendships (23, 24), whereas other studies found no significant relationship for these effects (25) or a negative relationship (26). Some recent studies suggest that this relationship was dependent on the internalizing behaviors involved. For example, a friend's somatization was linked to continued friendships at the end of the school year compared to friendships where neither member scored high on somatization. In same-sex friendships in which a member scored high on depression, a friendship breakup occurred more often by the end of the school year (27). The impact of clinically significant chronic pain on friendship stability is not known.

Friendships of ACP may be more primed to disruptions due to pain-related characteristics (e.g., school absence and canceling social plans) as well as poorer scores on indices of social-emotional well-being (e.g., loneliness, self-esteem, and depressed mood). Higher scores of positive friendship quality (e.g., social support from friends), as rated by one member of a same-sex friendship dyad that includes ACP, negatively impact the other dyad member's loneliness and depressed mood, suggesting that the role of friendship quality within friendships of ACP may be more complex (19). Surprisingly, negative friendship quality (e.g., conflict) was not associated with loneliness and depressed mood within the same friendships, but lower overall friendship satisfaction was associated with loneliness and depressed mood (19).

During mid- to late-adolescence, more exclusive trusting friendships exist. Yet, during this time, some healthy adolescent friendships change (28). Although ACP report the loss of friends since the onset of pain, it is unknown if their friendship breakup rates are higher than friendships among adolescents without pain. It is also unclear if the reasons for friendship breakups that do occur differ in adolescents without pain. The primary aim of this longitudinal study was to determine if friendships of ACP are less stable than those of non-pain adolescents over time and to explore the reasons for these friendship changes. The secondary aim of this study was to explore the factors (e.g., friendship features and social-emotional well-being) that were predictive of friendship stability, as well as to explore the impact of friendship breakups on the social-emotional well-being of ACP over time.

## Methods

A multiphase multisite cross-sectional project, including three distinct studies and inclusive of the same participants in all three studies, was conducted. Study 1 captured demographics and standardized measures to examine friendship feature differences on indices of social-emotional well-being between and within the friendship dyads when one adolescent has chronic pain, compared to non-pain dyads [refer to Forgeron et al. (19)] using a dyadic analysis approach as each member of the 62 dyads participated in Study 1. Individuals within the dyads that included an ACP had a stronger negative relationship between one's own loneliness and their perception of social support among their friendship network compared to controls (known as actor effects). They were also found to have partner effects, meaning that if the other member of the dyad rated their social support within their friendship network higher, it increased the actor's loneliness. This finding suggested that the friends of ACP had other friends with whom they could garner social support and companionship. Similar partner effects were found for perceived social support and depressed mood for dyads that included an ACP, but they were not found in controls. Study 2 examined the verbal and non-verbal exchanges between members of these same friendship dyads during the cold pressor pain task to determine how their friendship interactions were related to pain tolerance and pain intensity (29). Study 3, reported in this study, examined changes to friendship stability over time as well as changes in social-emotional well-being 1 year after they participated in the two previous lab-based studies. The present prospective longitudinal online survey was hosted on the Research Electronic Data Capture (REDCap) platform, which is a web application for building and managing online surveys and databases. Access to REDCap was carried out through one of the academic health centers that was part of this research, and thus, their information technology specialists and protocols secured the online data capture and database. An additional advantage of using REDCap was that it permitted the direct export of data into the data analysis software and generated reports on open-ended questions (30). The survey consisted of repeat measures from Study 1, along with three additional questions using branching logic to capture friendship stability.

In the current study, the following hypotheses were examined.

1. Participants who were part of a dyad that included an ACP member will have more friendship breakups compared to participants who were part of the friendship dyads without an ACP.
2. Participants who were part of the dyads that include an ACP member will have more negative friendship breakups (e.g., the dissolution of friendship due to an argument vs. no longer being in the same school) compared to participants who were part of the dyads without an ACP member.
3. Poorer scores on indices of social-emotional well-being at Time 1 (T1) and poorer scores on friendship features T1 will be associated with a friendship breakup at Time 2 (T2).
4. Adolescents with chronic pain will have poorer scores on indices of social-emotional well-being over time compared to those without chronic pain, controlling for scores at T1. Poorer outcomes on indices of social-emotional well-being will be more pronounced for ACP who experience a friendship breakup.

### Participants

Potential participants with chronic pain were recruited from one of the three participating Canadian pediatric health centers of chronic pain clinics. Prospective participants were given an invitation letter indicating that participation was voluntary and that if they wanted to participate, they would need to bring a same-sex friend of their selection to take part in the study. Non-pain friendship dyads were recruited using community advertisements in the three cities where the study took place. The community advertisement explicitly indicated that interested adolescents required a same-sex friend to take part in the study with them. Inclusion criteria for all adolescents were that they had to: (1) be between 13 and 18 years of age; (2) have the same-sex friend willing to participate; (3) read, write, and speak English; and (4) be in the correct grade for age. Additional inclusion criteria for the ACP were: (1) the presence of pain for at least 3 months and (2) persistence of pain despite treatment (not pain-free). Exclusion criteria for all adolescents were: (1) the presence of developmental delay; (2) the diagnosis of major psychiatric illness (e.g., schizophrenia and bipolar illness); and (3) the identified friend was a relative (e.g., cousin or sibling). An additional exclusion criterion for ACP was the presence of a life-threatening illness (e.g., cancer). Additional exclusion criteria for the non-pain adolescents were: (1) experiencing pain greater than once a month when interfered with activities or school attendance and (2) the presence of a chronic illness requiring medical care and interfering with activities or school attendance >1 day every month.

### Procedures

Participants, in their friendship dyads, attended one of the three labs at T1. During this lab visit (for Studies 1 and 2), they completed a battery of measures to capture demographic characteristics, friendship features, pain characteristics, school absence, and indices of social-emotional well-being (e.g., loneliness, depressed mood, self-esteem, and social anxiety) and participated in the cold pressor task. One year (12 months) after the lab visit (T1), an email was individually sent to each participant with the URL link to the follow-up survey (T2). All data were collected before the COVID-19 pandemic and related social distancing restrictions. Efforts to help improve retention included those recommended by Boys et al. (31) as they were successful in retaining 92% of adolescent participants at 18 months in a longitudinal study. These efforts included the collection of detailed contact information (emails, home phone numbers, and mailing address), incentives for participation ($10.00 Canadian to a national bookstore to complete T2 survey), and reminders. Participants in this study were contacted by email at 3, 6, and 9 months after the lab study visit to remind them of the upcoming survey and confirm their email addresses. If there was no reply to the 3-, 6-, or 9-month emails, participants were contacted using their home phone number to ensure their continued interest and confirm their email address.

### Measures

#### Demographic Data

A study-specific 6-item demographic form to capture age, sex, school grade, rural or urban community living, and the number of days of school absences for illness in the past month was completed by all participants at T1. Only the number of days of school absences for illness in the past month was captured again at T2.

#### Pain Assessment Form

At both T1 and T2, participants with painful conditions completed a form to capture pain location, frequency, and intensity at both T1 (lab visit) and T2 (1 year later). The *Numeric Rating Scale* (NRS-11) (32) was used to capture pain intensity (0–10) based on their average level of pain over the past 7 days. To capture pain-related social role disability, ACP also completed the 6-item *Pediatric Migraine Disability Assessment (PedMIDAS)* (33, 34). The PedMIDAS has a test–retest of 0.80 and internal consistency of 0.78 (33), and it has been used with similar pain populations in other studies due to the nature of the wording of the items that focus on social role interference (e.g., ability to do household chores) instead of the items focused more on physical role interference (e.g., how far can one walk). Additionally, the wording of the items was amendable to a single word change for this population by using pain instead of headaches (e.g., *How many days in the past month did you not participate in other activities due to pain (i.e., play, go out, sports, etc.)?*) (18, 35). The PedMIDAS has been recommended by the PedIMPACT consensus group to measure pain-related social disability in ACP (36) and has been used in several studies with diverse pain populations (18, 19). The PedMIDAS produces scores ranging from 0 to 260, which can be categorized by the degree of disability: 10 or less indicates little to no pain-related disability; 11–30 indicates mild pain-related disability; 31–50 indicates moderate pain-related disability; and 50 and above indicates severe pain-related disability (33, 34).

#### Friendship Features

All participants completed the two standardized measures to capture friendship features plus three additional questions at both T1 and T2. The *Friendship Quality Scale (FQS)* (37) is a 23-item 5-point self-report scale that captures five dimensions of friendship quality (companionships, conflict, help/support, closeness, and security) that are reducible to two main subscales; *positive* and *negative* friendship quality scores (higher scores for each reflect more positive or more negative quality) (38). In this study, the overall internal reliability of this measure was high at α = 0.814. The single factor 20-item self-report *Perceived Social Support-Friend (PSS-Fr)* (39, 40) was used to capture participants' perceived social support from all their friends within their social network with higher scores reflecting more support. The PSS-Fr has a test-retest reliability of 0.83 and internal consistency of 0.88. The internal reliability of this measure within this study was acceptable at α = 0.71. The *Ranking of Friendship* is a single item that asked participants to indicate the closeness of the friendship with the person who came to the lab with them (very best, best, close, friend, and acquaintance) (41), lower scores reflect a closer friendship. The last two friendship questions were both single items and used a 5-point Likert type scale to capture how satisfied one was with this friendship (1 = not very satisfied to 5 very satisfied), and how well one thought the friendship was going (1 not going well to 5 going very well). The use of these two additional friendship questions has been used in previous studies of typical friendships (41, 42). The inclusion of these two questions in this study was to explore ACP changes in friendship closeness, helpfulness of friends, and understanding from friends after the onset of chronic pain (7, 43).

#### Friendship Stability

Three questions were created to capture friendship stability. Using branching logic to decrease the survey burden, the first question asked a binary question about friendship changes. If the response was yes, the second question appeared and gave options to determine the type of change: did the friendship grow closer and grow apart due to some negative circumstances; or was the friendship change due to natural drift (e.g., different classes and moved away). The third question was an open-ended question for all those who experienced a friendship change to provide their perspectives on the reason for the friendship change.

#### Indices of Social-Emotional Well-Being

At both T1 and T2, the four indices of social-emotional well-being were assessed using standardized measures. Social anxiety was captured using the *Social Anxiety Scale for Adolescents* (44), which is a 22 item self-report instrument on which higher scores indicate increases in social anxiety. This measure can be used as an overall global score or scores based on the three subscales. Only the overall global score was used in this study. The test-retest reliability of the *Social Anxiety Scale for Adolescents* is 0.70, and internal consistency is 0.90 (44). The internal reliability of this measure in this study was α = 0.94. Participant loneliness was assessed using the *Loneliness Scale* (45, 46), which is a 24-item unidimensional self-report scale that consists of 16 loneliness questions and 8 filler questions, with higher scores indicating more loneliness. In previous research, the *Loneliness Scale* had internal reliability of 0.87–0.90 and in this study, the internal reliability was high at α = 0.94. The *Centre for Epidemiologic Studies Depression Scale (CESD)* (47) was used to capture depressed mood and consists of a 20-item unidimensional self-report measure where higher scores indicate a greater likelihood of depressed mood. The CESD has internal reliability of 0.87 and was found to predict depression based on diagnostic interviews (47). The internal reliability of the CESD in this study was α = 0.90. Finally, the *Rosenberg Self-Esteem Scale* was used to capture a global self-esteem score (48). This 10-item self-report measure asks participants to rate themselves on the 10 statements from 0 (disagree strongly) to 3 (agree strongly) with higher scores indicating higher self-esteem (48). The internal reliability of this measure in this study was high at α = 0.94.

### Analysis Plan

The analysis plan was based on the overall project, and thus, for the primary outcomes, we used a medium effect size in our sample size calculations. The first study [refer to (19)] used a 4 group × 4 factor design with the indices of social-emotional well-being (social anxiety, self-esteem, depressed mood, and loneliness) as the outcomes of interest, which required 80 participants (40 dyads). Power analysis with α = 0.05 and β = 0.20 using G^*^ Power 3 (48) resulted in the suggested sample size of 92 (46 dyads). To offset a potential loss to a follow-up for this prospective study, 25% more dyads were recruited for a total of 60 dyads (30 dyads with an adolescent with chronic pain and 30 controls). The follow-up analysis reported in this study was restricted to *n* = 83 and uneven group sizes. Using G^*^Power (49) for the 2 group × 2 time factor repeated-measures ANOVA analyses using a large effect size, the total sample size is 86. The logistic regression sample size calculation was based on the recommendations of Peduzzi et al. (50) using the formula (*n* = 10 *k/p*), which suggested a sample size of 83 for analysis with four predictor variables and the proportion of events (friendship breakups) of 0.48 and up to 166 for analysis with eight predictor variables. However, Vittinghoff and McCulloch (51) have noted that this ratio of observations per variable may be too conservative and suggest that as low as five observations per event may be acceptable. Additionally, as noted by Tabachnick and Fidell (52), cell size for the logistic regression analysis is “best if all expected cell frequencies are greater than one and that no more than 20% are less than five” (p. 442), which was met in the current analysis.

Debate exists as to the best analysis for dyadic data because of the potential of interdependence in dyad scores on predictor variables. In treating dyadic data as independent, suggestions differ as to the cut-off for interclass correlations (ICCs) for variables of concern and range from 0.45 (53) to 0.7 (42). The larger the ICC, the greater the potential bias in the analysis (either type I or II error, depending on the direction of the influence of the behaviors). The ICCs for friendship features at T1 were between 0.196 and 0.59 except for social support from one's friends, which was 0.925 [refer to Forgeron et al. (19)]. Moreover, because not all participants in each dyad took part in this 1-year follow-up study, the ICCs would decrease further and thus all participants were used in the analysis for maximum power.

Demographic data were analyzed using ANOVA for continuous variables and Chi-square for categorical. To answer Hypotheses 1 and 2, Chi-squared analyses were completed using dyad level membership as the grouping variable. Logistic regression was conducted to answer Hypothesis 3, and a series of 2 × 2 repeated measures ANOVAs were conducted to answer Hypothesis 4.

## Results

### Participants

Eighty-three participants completed the T2 study survey for a 68% response rate from the first study (T1). Of those 83 participants, 19 (22.9% of the sample size) represented one member of a dyad (meaning that another person in the dyad did not take part in this study) whereas the other 64 participants represented both members of 32 dyads. Although most participants (*n* = 49) represented members of the non-pain adolescent friendship dyads, 35 participants were members of dyads that included an ACP. The distribution of sex between non-pain dyad participants and participants from the dyads that included an ACP was not significant (*Fisher's exact test p* = *1.0)*. The overall mean age of the entire sample was15.29 years of age (SD = 1.26), and there was no significant age difference (*p* = 0.465) between the dyad types (refer to Table 1).

**Table 1:** Distributions of the sample by dyad type, sex, and age.

**Dyads**	**Female**	**Male**	**Age mean (SD)**
			**T1**
Pain dyad	29	5	15.41 years (0.22)
Non-pain dyad	41	8	15.20 years (0.18)

*Chi-square for the distribution of sex between pain and non-pain dyads, non-significant (Fisher's exact test p = 1.0); ANOVA for differences in mean age between the dyad types, non-significant (p = 0.465)*.

For participants with chronic pain who took part in this follow-up study (*n* = 19), most (73.6%; *n* = 14) had constant pain or daily pain with an average pain intensity on the NRS of 5.67 (SD 1.82). The pain locations of the ACP varied as follows: multiple pain sites 63.1% (*n* = 12); back 15.7% (*n* = 3); eyes 5.2% (*n* = 1); head 5.2% (*n* = 1); legs 5.2% (*n* = 1); and abdomen 5.2% (*n* = 1). There was a variation in the degree of pain-related disability among these participants with chronic pain with a T1 PedMIDAS mean score of 87.74 (SD 73.71) for all the ACP, indicating that most of the participants experienced significant pain-related disability. However, the T2 PedMIDAS mean score for the same participants was 48.47 (SD 54.50), suggesting that 1 year later the same participants now experienced moderate pain-related disability. Specifically, at T1, 57.8% (*n* = 11) of the participants with chronic pain experienced severe pain-related disability (score over 50) whereas this dropped to 31.5% (*n* = 6) at T2. The percentage of those who experienced little to no pain-related disability (score 10 or less) at T2 rose to 42.1% (*n* = 8) from 26.3% (*n* = 5) at T1. Overall, there was an improvement in the degree of some of the ACP of pain-related disability after 1 year.

Comparisons between the types of dyads (dyads with a chronic pain member compared to dyad without a chronic pain member) were conducted to determine if there were differences in friendship features at T1 and T2. There were no differences in the length of the friendship, ranking of the friendship, how satisfied they were with their friendship, or how well they thought that the friendship was going at T1 as well as at T2. However, there were significant differences at T1 and T2 between dyad types for negative friendship quality. Dyads that included an ACP had significantly lower scores for negative friendship quality at both T1 and T2 as well as significantly lower scores for positive friendship quality at T2 (refer to Table 2 for details of these findings).

**Table 2:** ANOVA for friendship features [interclass correlation (ICC) below 0.45 at T1].

**Friendship feature**	**Dyad with ACP**	**Dyad without ACP**	***F* value**	***p*-value**
	**M (SD)**	**M (SD)**		
T1 Friend years	7.09 (4.99)	5.62 (4.28)	2.00	0.161
T1 Friend ranking	1.59 (0.74)	1.8 (0.84)	1.34	0.250
T2 Friend ranking	2.76 (1.44)	2.33 (1.20)	2.06	0.155
T1 How well	4.65 (0.48)	4.76 (0.47)	1.46	0.229
T2 How well	3.7 (1.26)	4.1 (0.86)	2.99	0.087
T1 How satisfied	4.71 (0.46)	4.8 (0.41)	0.87	0.352
T2 How satisfied	3.85 (1.17)	4.06 (0.95)	0.813	0.370
T1 Neg fr quality	17.08 (5.722)	19.67 (4.44)	5.35	0.023^*^
T2 Neg fr quality	16.90 (4.77)	19.20 (4.22)	5.13	0.026^*^
T1 Pos fr quality	78.02 (9.02)	76.04 (8.05)	0.736	0.393
T2 Pos fr quality	58.87 (19.08)	69.04 (13.50)	7.78	0.007^*^
T1 PSS-Fr	17.38 (14.06)	15.59 (3.14)	Baseline ICC 0.96	
T2 PSS-Fr	21.84 (2.45)	20.66 (3.37)	Baseline ICC 0.96	

*T1, Time 1; T2, Time 2; M, Mean; SD, Standard deviation; Friend years, Length of time they have been friends in years; friend ranking (1 very best friend, 5 acquaintance), How close their friends is; How well, How well they think the friendship is going; How satisfied, How satisfied they are with the friendship; Neg fr quality, Negative friendship quality (of present friendship); Pos fr quality, Positive friendship quality (of present friendship); PSS-Fr (ANOVA not conducted on PSS-Fr due to high ICC at T1), Perceived social support from all friends*.

* *p value 0.05*.

There were only a few data points missing for a few participants and these were for the questions about friendship changes after 1 year. For example, one participant from a dyad that included an ACP and another from the control dyad did not answer regarding whether their friendship had changed. However, for these two participants the data revealed that both participants were from a dyad that included both members at T2 and the other member of the dyad, in both cases, indicated that their friendship was no longer as close. No attempts were made to input the data for these missing data on friendships. Therefore, when the analysis was presented with <83 responses, it indicated missing data on that variable. There were only four participants who indicated that their friendship grew closer during the year. This led to cell sizes with zero observations in some cases, and therefore, these participants were collapsed into the group who did not experience a friendship breakup. Specifically, three participants from friendship dyads without a chronic pain member and one participant from friendship dyads that included an ACP member who did not have chronic pain, indicated that their friendship grew closer during the year. Two of these participants were from dyads where both members participated (both of which were control dyads) and the other members of the dyad did not corroborate this increased closeness. In one case, the other member stated that the friendship was unchanged, and in another case, the other member of the dyad stated that they had drifted apart.

Data were inspected for normality, and variables at T2 were normally distributed based on skewness and kurtosis values, however, several of the variables of interest at T1 were not (e.g., CES-D scores and loneliness). Although not all variables at T1 were normally distributed, repeated-measures ANOVA analysis is robust against normality violations due to the skewness and kurtosis of variables; thus, we did not transform the variables (52, 54). Also, logistic regression is not based on a linear relationship, thus, the normality of the predictor variables is not necessary [p. 437 (52)].

### Bivariate Correlations

Spearman correlations are reported for the correlations that include a non-normally distributed variable and those with a relationship with a binominal variable. Having chronic pain was significantly positively correlated with worse outcomes on some indices of social-emotional well-being at T1 and T2. Being an ACP had a stronger positive correlation with the depressed mood at T1 (0.411^*^) compared to T2 (0.243^*^), whereas the positive correlation between being an ACP and having higher scores on loneliness was relatively stable over the year with the correlation for loneliness at T1 = 0.352^*^ and at T2 = 0.320^*^. Higher levels of social anxiety (T1 0.272^*^; T2 = 0.169) and lower scores on self-esteem (T1 = −0.260^*^; T2 = −0.165) were only significantly correlated with having chronic pain at T1. However, at T2, ACP scored the positive qualities of their friendship less favorably than participants without chronic pain (−0.234^*^). Moreover, having chronic pain was significantly correlated with experiencing a friendship breakup at T2 (*r* = 0.225^*^), as well as experiencing a friendship breakup due to a conflict (*r* = 0.239^*^). Pain-related disability scores at T1 were not significantly correlated with experiencing a friendship breakup (*r* = 0.062) or friendship breakup due to a conflict (*r* = 0.131), suggesting that pain-related characteristics other than disability contribute to the association between having chronic pain and a friendship breakup.

Bivariate correlations were completed to examine associations between indices of social-emotional well-being and friendship characteristics at both T1 and T2 and friendship stability at T2 (refer to Tables 3, 4). Friendship stability (a friendship breakup or no change in friendship at T2 was coded as 1 for breakup and 0 for no change) had a significant negative correlation with the T2 measures of how satisfied they were with the friendship (*r* = −0.583^**^), positive friendship quality (*r* = −0.671^**^), how well they thought the friendship was going (*r* = −0.651^**^), and a positive correlation with friendship for closeness [1 = very best friend; 5 = acquaintance [*r* = 0.650^**^]], indicating, as expected, that friendship features were negatively impacted by a friendship breakup. The only T2 measure of social-emotional well-being that correlated with friendship stability at T2 was depressed mood (*r* = 0.259^**^). Similarly, the only T1 index of social-emotional well-being that was associated with friendship stability at T2 was the participant's self-esteem (*r* = −0.241^*^). The only T1 friendship measure that had a significant bivariate correlation with friendship stability was the length of friendship (*r* = −0.229^*^), meaning that those with shorter friendships were more apt to experience a friendship break up at T2. Sex and age were not significantly associated with friendship stability or a friendship breakup due to a conflict. For those who had a friendship breakup due to a conflict at T2, the following T1 measures were significantly correlated: participant loneliness (*r* = 0.227^*^), depressed mood (*r* = 0.231^*^), and social anxiety (*r* = 0.275^*^). No T1 friendship features were correlated with a conflict breakup at T2. The T2 measures that were significantly correlated with a friendship breakup due to a conflict at T2 were positive friendship quality (−0.390^*^), negative friendship quality (−0.350^**^), pain-related disability (0.261^*^), ranking of friendship (*r* = 0.336^**^), how satisfied they were with the friendship (−0.455^**^), and how well they thought the friendship was going (−0.443^**^).

**Table 3:** Bivariate correlations T1 friendship features and social emotional well-being with friendship breakup.

	**Sex**	**ACP Dyad**	**Fr year T1**	**Fr break**	**Conflict**	**CES-D T1**	**RES T1**	**Lone T1**	**SAS T1**	**Fr rank**	**How well T1**	**Sat T1**	**PSSFr T1**	**FQS Neg T1**	**FQS pos T1**
ACP	0.002	0.654^**^	0.182	0.225^*^	0.239^*^	0.411^*^	−0.259^*^	0.352^*^	0.272^*^	−0.137	0.006	−0.028	−0.099	−0.134	0.024
Sex		−0.022	0.252^*^	−0.152	0.184	−0.170	0.216^*^	−0.143	−0.242^*^	0.078	−0.112	0.010	−0.072	0.130	−0.374^**^
ACP Dyad			0.158	0.207	0.219^*^	0.389^**^	−0.331^**^	0.364^**^	0.328^**^	−0.123	−0.158	−0.104	−0.076	−0.234^*^	0.130
Fr year T1				−0.229^*^	−0.010	0.191	−0.117	0.198	0.002	−0.214	0.098	0.015	0.003	−0.341^**^	0.021
Fr break					0.389^*^	0.206	−0.241^*^	0.087	0.207	0.009	−0.057	−0.108	0.007	0.196	−0.096
Conflict						0.231^*^	−0.164	0.227^*^	0.275^*^	−0.153	0.056	−0.051	−0.062	0.080	−0.031
CES-D T1							−0.650^**^	0.726^**^	0.595^**^	−0.120	−0.195	−0.300^**^	−0.393^**^	−0.142	−0.146
RSE T1								−0.624^**^	−0.682^**^	0.095	0.130	0.194	0.262^*^	0.149	−0.032
Lone T1									0.580^**^	−0.002	−0.071	−0.083	−0.477^**^	−0.223^*^	−0.212
SAS T1										−0.074	−0.085	−0.175	−0.371^**^	−0.146	−0.026
Fr rank											−0.267^*^	−0.376^**^	−0.088	−0.062	−0.476^**^
How well T1												0.497^**^	−0.088	0.174	0.430^**^
Sat T1													0.181	0.060	0.484^**^
PSSFr T1														0.137	0.291^**^
FQS Neg T1															0.260^*^

*T1, Time 1; ACP, Adolescent with chronic pain; ACP dyad, Membership in a dyad that includes an ACP; Fr year, How long have you been friends (in years); Fr break, Friendship breakup; conflict, Breakup in friendship due to conflict; CES-D, depression score; RSE, self-esteem score; Lone, loneliness score; SAS, social anxiety score; how close is the friendship (1 = very best friend; 5 = acquaintance); How well, How well do you think this friendship is going; Sat, How satisfied are you with this friendship; PSSFr, Perceived social support from all your friends; FQS neg, Negative friendship quality; FQS pos, Positive Friendship quality*.

* *p value 0.05,*

** *p value 0.01*.

**Table 4:** Bivariate correlations T2 friendship features and social emotional well-being with friendship breakup.

	**Sex**	**ACP Dyad**	**Fr year T1**	**Fr break**	**Conflict**	**CES-D T2**	**RES T2**	**Lone T2**	**SAS T2**	**Fr rank**	**How well T2**	**Sat T2**	**PSSFr T2**	**FQS Neg T2**	**FQS pos T2**
ACP	0.002	0.654^**^	0.182	0.225^*^	0.239^*^	0.243^*^	−0.165	0.320^**^	0.169	0.079	−0.173	−0.110	0.029	−0.115	−0.234^*^
Sex		−0.022	0.252^*^	−0.196	0.184	−0.237^*^	0.399^**^	−0.165	−0.317^**^	−0.169	0.165	0.161	−0.002	−0.147	0.035
ACP dyads			0.158	0.175	0.239	0.195	−0.151	0.279^*^	0.167	0.144	−0.141	−0.076	0.187	−0.260^*^	−0.259^*^
Fr year T1				−0.229^*^	−0.010	0.209	−0.073	0.137	0.100		0.184	0.135	−0.003	−0.099	0.116
Fr break					0.389^*^	0.299^**^	−0.190	0.189	0.047	0.650^**^	−0.651^**^	−0.583^**^	−0.156	−0.092	−0.671^**^
Conflict						0.270	−0.101	0.166	0.270	0.336^**^	−0.421^*^	−0.499^**^	0.006	−0.453^**^	−0.322^*^
CES-D T2							−0.723^**^	0.666^**^	0.558^**^	0.121	−0.310^**^	−0.264^*^	−0.032	−0.311^**^	−0.293^**^
RES T2								−0.692^**^	−0.677^**^	−0.106	0.284^*^	0.218	0.054	0.296^**^	0.270^*^
Lone T2									0.571^*^	−0.029	−0.175	−0.129	0.129	−0.270^*^	−0.216
SAS T2											−0.112	−0.182	−0.039	−0.284^*^	−0.165
How well T2												0.786^**^	0.120	0.276^*^	0.749^**^
Sat T2													0.192	0.313^**^	0.651^**^
PSSFr T2														−0.075	0.103
FQS Neg T2															0.321^**^

*T2, Time 2; ACP, Adolescent with chronic pain; ACP dyad, Membership in a dyad that includes an ACP; Fr year, How long have you been friends (in years); Fr break, Friendship breakup; conflict, Breakup in friendship due to conflict; CES-D, depression score; RSE, self-esteem score; Lone, loneliness score; SAS, social anxiety score; how close is the friendship (1 = very best friend; 5 = acquaintance); How well, How well do you think this friendship is going; Sat, How satisfied are you with this friendship; PSSFr, Perceived social support from all your friends; FQS neg, Negative friendship quality; FQS pos, Positive Friendship quality*.

* *p value 0.05,*

** *p value 0.01*.

### Friendship Features and Stability at 1-Year Follow-Up

The participants were asked if since coming to the lab 1 year ago their friendship had changed. Just over 50% (*n* = 42) of the participants indicated that their friendship had not changed while 48.1% (*n* = 39) indicated that they experienced a friendship breakup. There were significantly more participants from dyads with an ACP member (60.6%) who indicated a friendship breakup over the year (*X*^2^ = 0.051). Thus, we confirm our first hypothesis that dyads with an ACP member experience more friendship breakups 1 year later (refer to Table 5 for details).

**Table 5:** Chi-square for a change in friendship.

**Dyad types**	**Stable**	**Breakup**	**Total**	**Fisher's exact test (one-sided)**
Adolescent with	13	20	33	
chronic pain dyads	39.4%	60.6%		
Control dyads	29	19	48	
	60.4%	39.6%		
Total	42	39	81	0.051
	51.9%	48.1%		

*Adolescent with chronic pain dyads, Participant is a member of the dyad included an adolescent with chronic pain; Control dyads, Participant is a member of the dyad that did not include an adolescent with chronic pain; Stable, No change in friendship over the year; Breakup, No longer friends after 1 year*.

To answer our second hypothesis that ACP would experience a more negative friendship breakup at 1 year, a Chi-squared analysis was conducted to examine the frequency of the number of changes in friendships, negative friendship breakups, and non-conflict reasons for friendship breakups. Although 21.2% of the dyads with an ACP member experienced a friendship breakup due to a conflict compared to 6.3% of control dyads, the difference in these results was not significant (Pearson two-sided *X*^2^ = 0.067) (refer to Table 6). Nevertheless, to further understand the reasons for friendship breakups, an open-ended question was included in the survey. The reasons for negative friendship breakups included situations like this one provided by a male ACP participant: “*The friend I came with ended up bullying me. It was all verbal. We are trying to work it out. But it is hard. He was really mean, and I am having trouble forgiving and moving past it. He says he is sorry and has not done it [again] in 6 months, but words hurt*.” A female ACP participant wrote that her friendship changed, “*Because she was not there for me after something terrible happened, and she picked another friend over me*.” Similarly, a female member of a control dyad indicated that “*We got in a very big fight and did not speak for almost 2 months*.” Examples of reasons that they grew apart in both dyad types were like this one given by a female participant from a control dyad, “*We don't go to the same school anymore, so we don't see each other near as much*.” The main reason cited for non-conflict friendship breakups was a decrease in physical proximity. However, there was an ACP who stated that “…[we] *haven't been able to spend as much time together. I'm not always able to see them because my health prevents me from doing so*.”

**Table 6:** Chi-squared analysis for friendship breakup reasons.

**Dyad type**	**Stable**	**Conflict breakup**	**Non-conflict breakup**	**Total**	**Person *X*^**2**^** **(2 sided)**
Adolescent with	13	7	13	33	
chronic pain dyads	39.4%	21.2%	39.4%		
Control dyads	29	3	16	48	
	60.4%	6.3%	33.3%		
Total	42	10	29	81	0.067
	51.9%	12.3%	35.8%		

*ACP dyads, Participant is a member of the dyad included an adolescent with chronic pain; Control dyads, Participant is a member of the dyad that did not include an adolescent with chronic pain; Stable, No change in friendship over the year; Conflict breakup, No longer friends after 1 year due to a conflict; Non-conflict breakup, No longer friends after 1 year but not due to a conflict*.

### Social-Emotional Well-Being and Friendship Features at T1 Predict Friendship Stability at T2

A logistical regression analysis was conducted to explore whether chronic pain, social-emotional well-being, and friendship features at T1 predicted friendship stability at T2. Chronic pain, the length of friendship, and T1 self-esteem were the only T1 scores that were correlated with friendship stability at T2 and, therefore, were entered into the model (refer to significant correlations mentioned above). Despite sex not having a significant correlation with the outcome, it was entered into the equation for theoretical reasons. Overall, the model explained almost 23% of the change in friendship stability. Change in friendship was coded 1 and no change was coded 0, thus for every 1 unit increase in the length of friendship the odds of experiencing a friendship breakup dropped by 0.868 times. However, if the participant was an ACP, the odds of experiencing a friendship breakup were 3.981 times the odds of not experiencing a friendship breakup. The score of self-esteem at T1 as well as sex was not significant in the model. Therefore, shorter friendships and experiencing chronic pain increased the odds of having a friendship breakup at T2 (refer to Table 7).

**Table 7:** Logistic regression: predicting friendship breakups from friendship and social-emotional well-being factors.

**Predictor**	**Unstandardized B weight**	**SE**	**Wald**	***df***	**Sig**	**Exp(B)**	**95% CI**
Constant	2.142	1.156	3.431	1	0.064	8.517	
Sex	−0.557	0.766	0.568	1	0.451	0.561	0.846–1.023
T1 RSES	−0.072	0.0.49	2.02	1	0.138	0.930	0.067–0.994
T1 Fr years	−0.142	0.060	5.627	1	0.018	0.868	0.772–0.976
ACP	1.382	0.659	4.388	1	0.036	3.981	1.093–14.499

*Nagelkerke R^2^ for the model = 0.230; B weight, Beta weight; SE, Standard error; df, Degrees of freedom; Sig, Significance statistic; Exp(B), Odds ratio; CI, Confidence interval; Sex, (0 = male; 1 = female); T1 RSES, Time 1 self-esteem score; T1 Fr sat, Time one friendship satisfaction score; T1 Fr years, Time one length of friendship in years; ACP, Adolescents with chronic pain*.

### ACP Will Have Poorer Social-Emotional Well-Being Over Time (Controlling for T1 Measures) Compared to Non-Pain Participants, and This Will Be More Pronounced for ACP Who Experience a Friendship Breakup

A series of four repeated-measures ANOVAs (2 × 2 design) was conducted to examine the effects of a friendship breakup on indices of social-emotional well-being for ACP compared to non-pain participants. Although group sizes differed, the Box *M* statistics for each of the repeated-measures ANOVAs was not significant at *p* > 0.01 and Pillai's statistic was used to evaluate multivariate significance [p. 252 (52)]. There were no significant changes to the rate of loneliness scores within subjects over time, but there was a significant difference in the rate of loneliness scores over time for those who had chronic pain (*F* = 8.406; *df* = 1; *p* = 0.005). Over the year, those with chronic pain became less lonely by ~7 points. Nevertheless, despite improvements in their loneliness, those with chronic pain continued to be lonelier compared to non-pain participants (*F* = 15.446; *df* = 1; *p* < 0.001) by almost 12 points on average. A friendship breakup did not have significant interaction with those having chronic pain or with loneliness over time.

Within-subject changes in one's scores on the depressed mood measure were not significantly different over time, regardless of whether the participant had chronic pain or not. However, between-subject findings indicated that ACP continued to have higher scores (~7 points higher) on average over time on depressed mood compared to participants without chronic pain (*F* = 8.262; *df* = 1; *p* = 0.005).

There were no within-subject differences with respect to changes in social anxiety scores over time regardless of whether one experienced a friendship breakup or not, and there were no statistically significant interactions. However, within-subject differences for scores on social anxiety approached significance over time if one had chronic pain (*F* = 3.816; *df* = 1; *p* = 0.054), suggesting that over time, those with chronic pain trended toward a less social anxiety score (by just over three points) over the year. Nevertheless, despite this trend toward an improvement in their social anxiety scores, on average, those with chronic pain scored significantly higher over the year than those without chronic pain by ~7.5 points (*F* = 4.48; *df* = 1; *p* = 0.037).

Finally, there were no within-subject effects on self-esteem scores over time, regardless of whether there was a friendship breakup or if one had chronic pain. However, there continued to be a significant difference in the average scores of self-esteem over time between groups such that ACP on average rated themselves three points lower on self-esteem (*F* = 4.64; *df* 1; *p* = 0.03). Unfortunately, a separate analysis of the impact of conflict compared to non-conflict friendship breakup and how this type of friendship breakups may impact indices of social-emotional well-being could not be conducted due to the small cell size across these two types of friendship breakups and group membership.

## Discussion

This study examined the friendship stability of ACP compared to friendships of non-pain adolescents after 1 year as well as the effects of friendship stability on social-emotional well-being. Friendship breakups during adolescence have been linked to multiple factors, including the degree of negative friendship quality (e.g., blaming friends and not helping friends in need) (37, 55), level of depressed mood (27), and somatization (26). However, non-clinical anxiety (24) and somatization (27) have predicted greater friendship stability. Somatic complaints have also been found to have no significant association with friendship stability (25). Informed by these studies and those on friendship experiences of ACP, several hypotheses were tested. A few but not all hypotheses were supported.

The hypothesis that participants who were members of a dyad including an adolescent with chronic pain would experience more friendship breakups by the end of the year (T2) compared to controls was supported. Despite the finding of dyads with an ACP experienced a 3-fold higher percentage in friendship breakups due to a conflict, this difference was not statistically significant. ACP have reported in qualitative studies (7, 9, 43) that they have lost friends since the onset of pain, and this present study provides quantitative evidence to support those reports, indicating that the best friend loss is not limited to a few ACP. This is troubling as participants rated their participating friend at T1 as their best or very best friend (19). Losing one's closest friend within a year could have implications for increased social isolation by ACP as they have been found to have fewer friends (6) and described distancing themselves from social situations (7, 9, 14). Given that by mid-adolescence, most identify their best friend as the main form of their social support (2, 3), increased rates of the best friendship breakups may result in ACP not receiving the social support they require, which may contribute to their continued higher levels of loneliness and depressed mood compared to controls. Clinicians should regularly assess the friendship status among ACP so that they can discuss strategies to establish and strengthen friendships for those who may be experiencing friendship difficulties and losses.

Unfortunately, it is not solely friendship loss that may be problematic. Some ACP reported not only the loss of friendship since the onset of pain but also that the treatment by these one-time closest friends was hurtful, and for some, reached levels of relational victimization (7). Some ACP in this study described the reasons for friendship breakups in terms of rejection. Although some of the non-pain participants cited similar reasons for a friendship breakup, it remains unknown if friendship breakups due to a conflict have a more detrimental effect for ACP as differing effects have been found for ACP compared to peers in other types of social conflict. For example, Fales et al. (56) found that ACP were not victimized more often than peers, but these hurtful experiences resulted in a greater negative impact on the depressed mood of ACP compared to peers. Research also suggests that some adolescent friendship breakups due to a conflict may transform into antipathetic relationships (57). In our study, a friendship breakup due to a conflict was associated with higher rates of loneliness, depressed mood, and pain-related disability at T2. We do not know what form the friendships that broke up due to a conflict in this study took and if higher pain-related disability, loneliness, and depressed mood were associated with antipathetic relationships. However, Higginson et al. (58) found that young people with chronic pain were reticent to disclose their pain condition to new friends, citing previous negative adolescent friendship experiences, leaving these young people challenged to garner pain-related social support, suggesting that negative friendship experiences for ACP are long-lasting. Therefore, research is needed to examine the effects of friendship breakups due to a conflict on the pain-related disability and social-emotional well-being of ACP, as well as the impact these experiences have on establishing future supportive friendships.

A friendship breakup at 1 year was predicted when the length of the friendship was shorter and when the participants experienced chronic pain at T1. Longer friendships may protect against friendship breakups, even in the face of health adversity (e.g., chronic pain), as they have long shared histories. Additionally, multi-context friendships have been found to be more stable over time than friendships that are mostly based on a single context (e.g., only school friends and only sports friends) (28). Perhaps, long shared history between friends provides time to develop more platonic affection, increase companionship, and evolve beyond proximity (e.g., same school and same class). Since quality friendships have been associated with decreases in somatic complaints (26), a strength-based approach to working with ACP may offer clinicians the strategies to help youth to maintain and strengthen best friendships over time (e.g., initiating contact, spending time together outside one venue, empathy for typical adolescent challenges, and decreases in pain-related behaviors balanced with disclosure).

Similar to research among typically developing adolescents, non-clinical rates of anxiety (24) did not increase the rate of friendship breakups nor were elevated rates of depression predictive of friendship breakups (24, 27). However, anxiety and somatization did not increase the rate of friendship stability in this study. Previous studies on somatization did not include adolescents with clinically significant symptoms. This study recruited participants with pain from tertiary centers, and therefore their level of pain and associated concerns may be significantly different compared to studies on somatization that included the complaints of headache, stomachache, and other symptoms such as nausea and fatigue. Perhaps, somatization is helpful in friendships up to a certain level as disclosure is a manifestation of closeness but when the voiced complaints continue and or interfere with social function, they become detrimental to ongoing closeness among friends. Although the friends of ACP have been included in this study, future studies are needed to better understand the perspectives of friends of ACP to have a deeper understanding of the influence of chronic pain-related factors (e.g., pain expression) on friendships.

Differing rates of loneliness were not associated with a friendship breakup at 1 year, thus this hypothesis was not supported. ACP had a decrease in their loneliness over the year, but it remained unclear if this change resulted in clinical improvements in loneliness as the measure did not have a clinical cut-off score. For ACP in long-term friendships, an additional year of having the same best friend may result in decreases in intimate loneliness (e.g., attachment to another like the best friend) compared to relational loneliness (e.g., feeling that one is lacking a social network like a peer group) (59). Unfortunately, ACP continued to experience higher rates of loneliness over time compared to peers. Loneliness during adolescence has been linked to poorer health outcomes in early adulthood (60), making it urgent to study loneliness among ACP. Loneliness was assessed in this study with a global measure. It would be helpful for future studies to capture loneliness using measures to separate relational and intimate loneliness.

Finally, there were no within-subject differences for the level of self-esteem or social anxiety in the repeat ANOVA models, meaning that ACP scores did not change over time any more than the scores for those without chronic pain. On a more positive note, there was an improvement in the degree of pain-related disability among the ACP over the year, but this was independent of friendship stability. All the ACP participants were recruited from tertiary pain clinics. Thus, improvements in pain-related disability and to any of the indices of social-emotional well-being could somehow be associated with any treatments that they may have received.

### Limitations

Limitations of this study included recruiting fewer males than females, precluding a subanalysis of male friendships. Therefore, these results must be interpreted with caution for male ACP. However, there were no significant differences in the number of male between groups. Moreover, sex did not have a significant correlation with any of the dependent variables. Not all participants who took part in T1 took part in the follow-up survey at T2, and therefore it is unknown if those who did not take part had a more friendship breakup. Finally, although based on the recommendations for sample size estimates needed for the logistic regression analysis, the sample size was <100 participants, which Long (61) suggests may increase type II errors, and therefore, the result from the logistic regression should be interpreted with caution.

## Conclusion

In conclusion, dyads that include an ACP experience friendship breakups more often than controls, and some of these friendship breakups were due to a conflict. The length of friendship and experiencing chronic pain predicted friendship stability, therefore, an understanding of what contributes to stable long-term friendships among ACP may help inform strategies to maintain and improve friendships for ACP who experience social challenges. Clinicians need to regularly assess the friendship status among ACP as the best friendship loss leaves ACP vulnerable to decreases in social support and other protective features of friendships. ACP continued to experience worse scores on loneliness and depressed mood compared to those without chronic pain. Studies are needed to understand the underlying reasons for worse scores among ACP as loneliness and depressed mood are linked to poorer long-term health. Research that examines the effects of friendship breakup due to conflict are also needed as this type of friendship dissolution may have differing effects for ACP.

## Data Availability Statement

The raw data supporting the conclusions of this article will be made available by the authors, without undue reservation.

## Ethics Statement

'The studies involving human participants were reviewed and approved by the Ethics Board of the Children's Hospital of Eastern Ontario; The University of Ottawa Research Ethics Board, The Research Ethics Board of the IWK Health Centre, and the University of Alberta Research Ethics Board. Older adolescents provided consent and younger adolescents provided assent with consent from their parent or legal guardian.

## Author Contributions

All authors listed have made a substantial, direct, and intellectual contribution to the work and approved it for publication.

## Funding

An Early Career Investigator Pain Research Grant from the Canadian Pain Society with the support of the Pfizer award to PF funded this study.

## Conflict of Interest

The authors declare that the research was conducted in the absence of any commercial or financial relationships that could be construed as a potential conflict of interest.

## Publisher's Note

All claims expressed in this article are solely those of the authors and do not necessarily represent those of their affiliated organizations, or those of the publisher, the editors and the reviewers. Any product that may be evaluated in this article, or claim that may be made by its manufacturer, is not guaranteed or endorsed by the publisher.
